# The growing interdisciplinarity of developmental psychopathology: Implications for science and training

**DOI:** 10.1017/S0954579424000580

**Published:** 2024-03-22

**Authors:** Ian H. Gotlib, Jessica L. Buthmann, Jessica P. Uy

**Affiliations:** Department of Psychology, Stanford University, Stanford, CA, USA

**Keywords:** developmental psychopathology, early adversity, interdisciplinarity, prevention, training

## Abstract

The field of developmental psychopathology has grown exponentially over the past decades, and has become increasingly multifaceted. The initial focus on understanding abnormal child psychology has broadened to the study of the origins of psychopathology, with the goals of preventing and alleviating disorder and promoting healthy development. In this paper, we discuss how technological advances and global events have expanded the questions that researchers in developmental psychopathology can address. We do so by describing a longitudinal study that we have been conducting for the past dozen years. We originally planned to examine the effects of early adversity on trajectories of brain development, endocrine function, and depressive symptoms across puberty; it has since become an interdisciplinary study encompassing diverse domains like inflammation, sleep, biological aging, the environment, and child functioning post-pandemic, that we believe will advance our understanding of neurobehavioral development. This increase in the breadth in our study emerged from an expansion of the field; we encourage researchers to embrace these dynamic changes. In this context, we discuss challenges, opportunities, and institutional changes related to the growing interdisciplinarity of the field with respect to training the next generation of investigators to mitigate the burden of mental illness in youth.

Fifty years ago, Achenbach (1974) described developmental psychopathology as a field that “hardly exists yet” (p. 3). Indeed, if the term “developmental psychopathology” was used at all, it was synonymous with Abnormal Child Psychology. Recognizing that this equivalence was likely to limit research in developmental psychopathology to the study of children, Sroufe and Rutter (1984) offered a definition of developmental psychopathology as “the study of the origins and course of individual patterns of behavioral maladaptation, whatever the age of onset, whatever the causes, whatever the transformations in behavioral manifestation, and however complex the course of the developmental pattern may be” (p. 18). Sroufe and Rutter noted further that the focus of developmental psychopathology is on understanding processes that underlie both continuity and change in patterns of adaptation. Just over 20 years later, Masten (2006) extended Sroufe and Rutter’s definition, adding that a goal of developmental psychopathology is “to prevent or reduce psychopathology and alleviate the burden of suffering it brings to individuals, families, and communities, while at the same time promoting healthy behavior and development” (p. 52).

To be sure, we have made significant progress in developmental psychopathology since Achenbach’s (1974) bleak assessment of the field; indeed, researchers have worked diligently to address Masten’s (2006) objectives concerning prevention and promotion. Importantly, however, as in other areas of science, advances in technology and data analysis over the past two decades have expanded the questions and the range of issues researchers are able to address in the field of developmental psychopathology. In particular, advances in genetics and neuroimaging, in multivariate statistical and analytic techniques, in diagnostic approaches, including new versions of the Diagnostic and Statistical Manual and new developments involving the National Institute of Mental Health (NIMH)’s Research Domain Criteria (RDoC), combined with the recent availability of large datasets that allow researchers to take a ‘big-data’ approach to address critical issues in developmental psychopathology, have informed (and have broadened) both the questions that investigators can ask and the methods they can use to address these new questions. In addition, recent events such as the COVID-19 pandemic and a renewed focus on climate-related issues have also led researchers in developmental psychopathology to raise and address novel questions.

At the same time that these advances and events have provided the impetus for investigators to pursue new research directions within developmental psychopathology, it is important to recognize that they also have significant implications for training in this field. Specifically, the stronger interdisciplinary focus in developmental psychopathology driven by this movement means that, as Masten (2006) foreshadowed, individual investigators are unlikely to be able to master the extensive background, approaches, methods, and analytic techniques that are becoming increasingly important in the integrative study of development across both disciplines and diverse domains of functioning, or to fully apply the obtained knowledge to practice and policy.

Our goal in this article is to articulate what we believe are important issues in developmental psychopathology; we do so in the context of describing the multidisciplinary evolution of a project that we have been conducting in our laboratory over the past ten years. We initially designed this project to examine the effects of early life stress (ELS) on trajectories of brain function/structure and of clinical symptoms in boys and girls over adolescence. Since beginning to conduct this study, however, we broadened our focus to address important questions, issues, and opportunities that arose and captured our interest mid-study. We describe these specific extensions to the initial aims of the project in greater detail below, discussing the impetus for these additions and presenting our belief that it is critical to the development of this field that researchers be open to leveraging and extending existing data to address emerging scientific questions.

We begin by describing our longitudinal study of the effects of ELS on psychobiological functioning across adolescence, briefly presenting the rationale and justification for conducting this investigation. We use this description of our project as a foundation for contextualizing important goals, issues, and methodological approaches in the field of developmental psychopathology, including the study of individuals through sensitive developmental periods, the use of longitudinal designs with repeated assessments to assess developmental changes and the emergence of psychopathology, and the integrative multi-domain examination of mechanisms and risk and protective factors for disorder across adolescence, in the service of generating and testing hypotheses relevant to human development. We discuss the ways in which this study has allowed us to examine a number of distinct questions in developmental psychopathology and, perhaps more importantly for this article, how we leveraged the longitudinal data we had collected to address new questions that emerged during the course of our study as relevant data became available in the public domain and as both the COVID-19 pandemic and a renewed focus on climate-related issues became salient; we describe how we responded to these events and issues by expanding the scope of our investigation in what we believe are important new directions. Finally, we conclude by describing challenges presented by the increasingly interdisciplinary nature of the field of developmental psychopathology for both mentors and mentees.

## Our ELS project

About ten years ago, in 2013, we initiated a longitudinal study designed to examine the effects of ELS on trajectories of brain structure, function, and connectivity, and their possible mediation of the association of ELS with depressive symptoms, in boys and girls over the transition through puberty. A major impetus for this study was the increasing prevalence of both early adversity and depressive symptomatology in adolescents over the past three decades. Although researchers had demonstrated that ELS is a significant risk factor for the development of a range of psychiatric symptoms in adolescents (LeMoult et al., 2019; McLaughlin et al., 2012), the mechanisms through which early adversity confers risk for psychopathology were (and still are) not well understood. Investigators also were unable to adequately explain the reasons underlying the well-documented sex difference in rates of depression during adolescence (Breslau et al., 2017). At the time we were designing our study, there were small but growing literatures indicating that ELS affects not only brain structure and function, particularly in regions involved in the generation and regulation of affect (Gee et al., 2013; Goff & Tottenham, 2015), but also neurobiological responses to stress (Hanson et al., 2016; Lupien et al., 2009). Given these findings, we posited that adverse experiences in childhood contribute to vulnerability for the development of mental health difficulties in adolescence by modifying neural pathways implicated in emotional functioning and stress responsivity and, further, that ELS has different effects on trajectories of brain development and subsequent psychopathology in girls than in boys. Finally, because we wrote the initial grant proposal for this study in response to a call for studies to assess the NIMH’s Research Domain Criteria (RDoC), we focused on the RDoC constructs of *Responsiveness to Reward and Responses to Sustained Threat*, examining how ELS affects brain circuitry and endocrine functioning implicated in these constructs (Insel et al., 2010).

**Figure 1.**
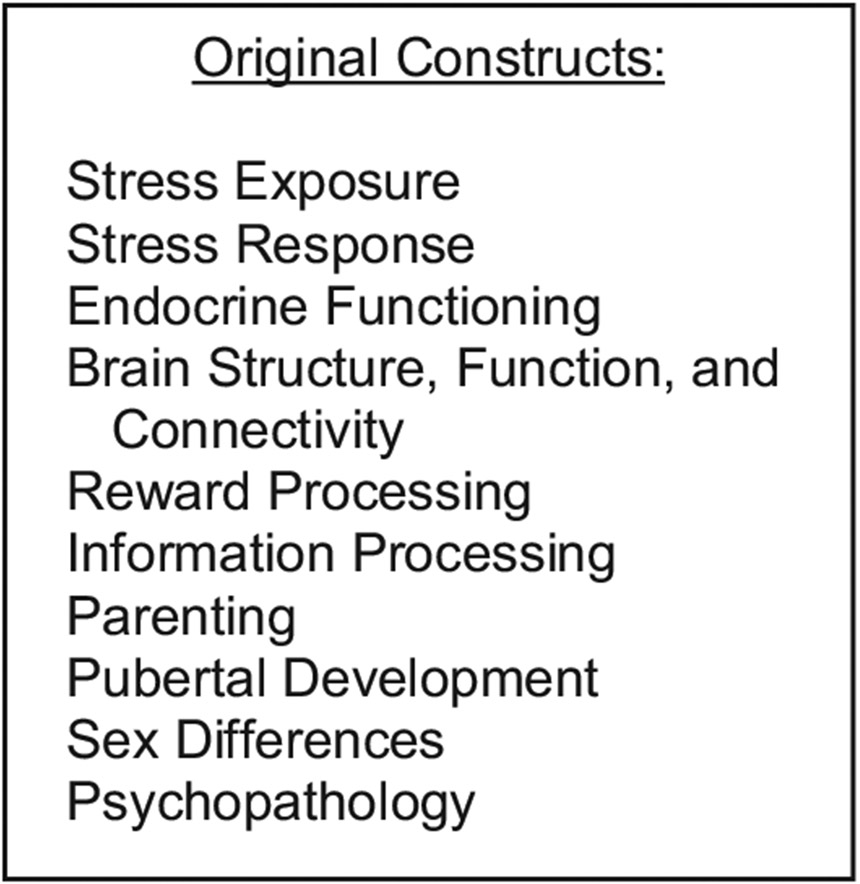
Constructs originally assessed in our early life stress study.

For this project we recruited a sample of 220 boys and girls in early puberty, between the ages of 9 and 13 years. We matched boys and girls on pubertal stage, which meant that boys were slightly older than girls at entry to the study. We proposed to assess the effects of ELS on brain function, structure, and connectivity, endocrine functioning, information-processing biases, and clinical symptoms relevant to the RDoC Reward and Threat constructs – initially at two timepoints separated by two years, then, in a renewal of our grant, at two additional timepoints, and finally, in a subsequent grant renewal, at a fifth timepoint when the participants reached 20 years of age. An overview of the constructs we proposed to assess in the original study is presented in Figure 1.

We have published several papers describing findings from this project that address the initial goals of our study. For example, in an early paper from this project Humphreys et al. (2016) reported that greater exposure to ELS was associated with attentional biases away from fearful facial expressions, which mediated the relation between ELS and poorer social functioning. In subsequent papers we described the adverse effects of ELS on endocrine functioning, including both stress-related and diurnal patterns of cortisol secretion (e.g., King et al., 2017; Kircanski et al., 2019), on neural function (e.g., Colich et al., 2017), structure (e.g., Ho et al., 2017; Humphreys et al., 2019; King et al., 2020), and connectivity (e.g., Borchers et al., 2022; Chahal et al., 2022), and on clinical functioning (e.g., LeMoult et al., 2019; Lee et al., 2023). In examining the effects of ELS on adolescents’ functioning, we also documented sex differences in associations of ELS, pubertal status, and brain metrics (e.g., Chahal et al., 2021; Colich et al., 2017).

In conducting these analyses, we were guided by, and largely followed, the analytic plan that we proposed in the grant to address and test specific questions and hypotheses. In doing so, we have been able to make what we believe are important contributions to our knowledge of how early adversity increases children’s and adolescents’ risk for developing psychopathology by altering stress-related endocrine function and stress- and reward-related brain function, structure, and connectivity; further, we have documented differential effects of sex and of earlier vs. later puberty on the psychobiological functioning of children and adolescents.

**Figure 2.**
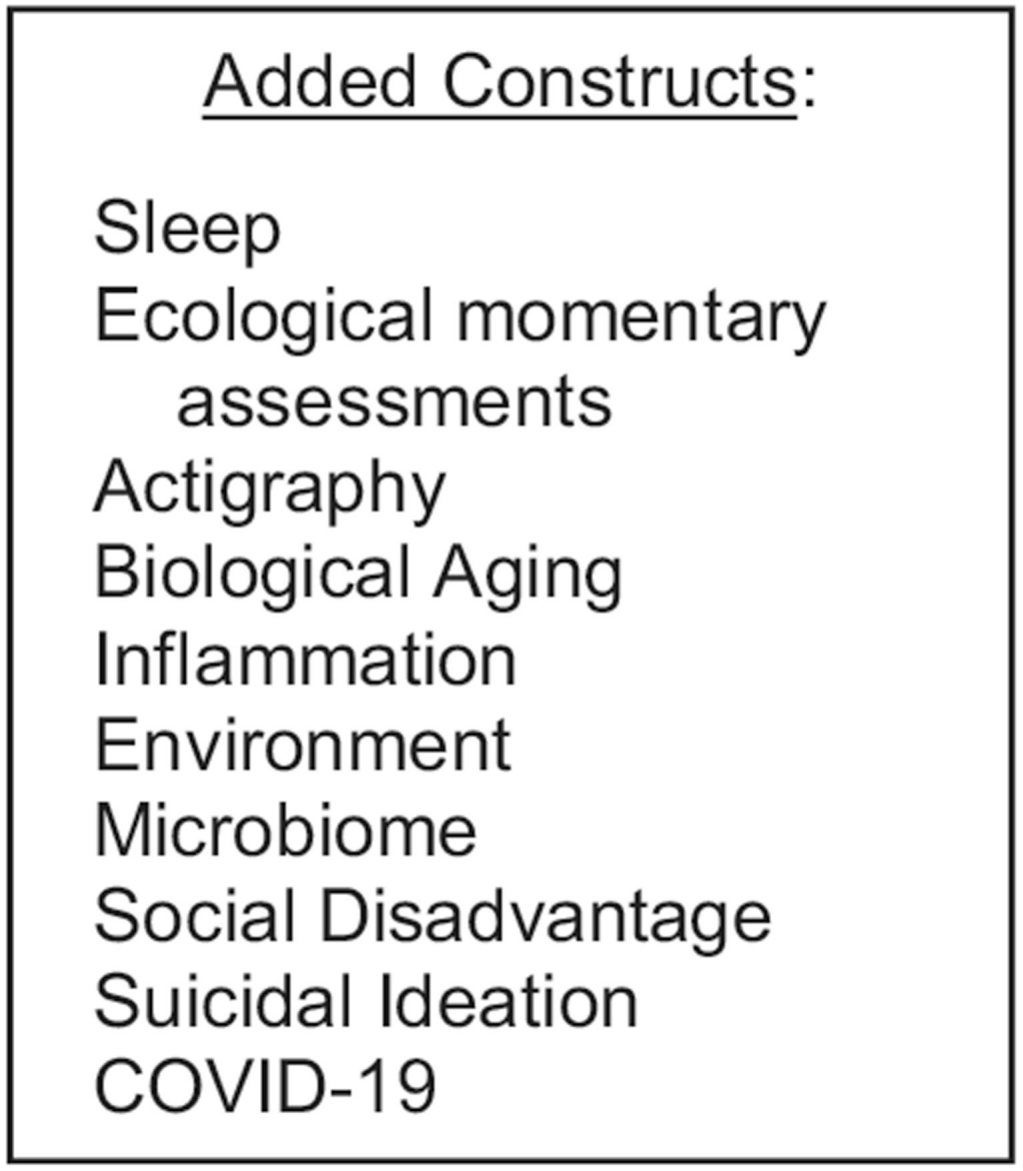
Constructs added to our early life stress study.

In planning and executing our proposed study, we attempted to address and adhere to contemporary principles and aims of developmental psychopathology. Certainly, it is important that researchers generate theory-driven hypotheses concerning issues in developmental psychopathology and design studies that will allow them to conduct the strongest tests of their formulations. Indeed, we followed these directives in designing and conducting our ELS project. At the same time, however, we believe that investigators must be willing and ready to adapt or extend their study to address societal and environmental issues that arise, as well as scientific advances that are reported, as they are conducting their planned research. In the sections below, we describe how we responded to issues, events, statistical advances, and new scientific foci that emerged as we continued to meet the goals of our project that we had originally proposed. More specifically, as novel data began to be reported concerning the effects of biological aging, inflammation, sleep, environmental toxins, and neighborhood socioeconomic disadvantage on adolescents’ functioning, we revised our experimental protocol and procedures to leverage our existing data to take advantage of these advances. For example, we began to obtain blood spots to assess inflammatory markers, administered ecological momentary assessment prompts and used actigraphy to assess sleep metrics, and collected saliva samples to assess aspects of biological aging (e.g., telomere length, DNA methylation) and microbiome composition and functioning. We also located and obtained access to environmental databases so that we could link our participants’ addresses to levels of air and water contaminants, allowing us to examine associations of exposure to environmental toxins with trajectories of both brain development and psychopathology. And as investigators began to report the development and utilization of increasingly sophisticated statistical methods, we revisited the analytic strategies we had proposed to use with our data and started to incorporate these state-of-the-art approaches into our project. Finally, as was the case for virtually all laboratories, our research was interrupted (and in many ways halted) by the COVID-19 pandemic and its associated lockdowns, requiring us to adapt quickly to constraints on conducting in-person human subjects research and to a changing work environment. In Figure 2 we present an overview of constructs we have added to our ELS study since we initiated the project.

## The increasing interdisciplinarity of developmental psychopathology

Before we describe in greater detail how we adapted our ELS project in response to the occurrences and advances we noted above, we want to comment more broadly on the growing interdisciplinary focus of research in the field of developmental psychopathology. Over the past two decades, developmental psychopathology has become exponentially more interdisciplinary. Investigators are making significant progress in this field by integrating advances in genetics, neuroimaging, and clinical science into their studies of child and adolescent development. Interestingly, this growing interdisciplinarity in developmental psychopathology was predicted fifty years ago by Cicchetti, Rutter, Sroufe, and Masten, all of whom recognized that different disciplines had an interest in various aspects of this field and in addressing different, but relevant, questions. Summarizing these perspectives, Cicchetti and Rogosch (2002) wrote, “Developmental psychopathology is an integrative discipline that seeks to unify, within a developmental, lifespan framework, contributions from multiple fields of inquiry with the goal of understanding psychopathology and its relation to normative adaptation …” (p. 7). These investigators also acknowledged, however, that there had thus far been little communication or interaction among these fields and disciplines, but all expected that this would increasingly become the norm.

It is important to note here that at the time these researchers were predicting a growing interdisciplinarity in developmental psychopathology, some of the scientific fields and areas of inquiry that have become integrated into research in developmental psychopathology had not yet even emerged. At that point, the important integration focused on reconciling perspectives from academic and clinical psychology; now, of course, the number, breadth, and diversity of scientific areas of study that have begun to (and should) be integrated into the field of developmental psychopathology are much greater. For example, it is now commonplace for studies of developmental psychopathology to include measures of neural structure, function, and connectivity, endocrine function, inflammation, and genetics; less common, but increasing in frequency, are investigations that integrate metrics of epigenetics and aging, the microbiome, and the physical environment, including air and water pollution, access to green spaces, and neighborhood crime and disadvantage. In the sections below, we describe why and how we adapted our research to include many of these emerging constructs of interest.

## Biology and developmental psychopathology

One of the most significant advances in developmental psychopathology over the past few decades is the integration of biology into this area of research. With advances in technology, incorporating biological markers into psychological research has become more affordable, feasible, and accessible to researchers in the social sciences. There are now growing literatures addressing how early environment and experiences interact with and shape biological aspects of human functioning that, in turn, affect the onset, severity, and persistence of psychopathology across development. These biological factors include genetics and epigenetics, biological aging (commonly indexed by telomere length, mitochondrial DNA, advanced pubertal stage), hypothalamic-pituitary-adrenal axis functioning (typically indexed by cortisol levels – diurnal cortisol, stress-reactivity cortisol, and hair cortisol), brain function, structure, and connectivity, and immune functioning. Foundational research in these areas has demonstrated that the mind and psychological health are not easily dissociable from the body and physical health, and that to understand, prevent, and treat psychopathology it is critical that we appreciate both mental and physical manifestations of diseases and their comorbidities.

In our study we had already proposed to obtain saliva samples to assay both stress-related and diurnal patterns of cortisol secretion (e.g., King et al., 2017; Kircanski et al., 2019) as well as sex hormones like dehydroepiandrosterone and testosterone (King et al., 2020). Given advances in the study of biological aging in developmental psychopathology, we leveraged these samples to examine both cross-sectional and longitudinal associations between depressive symptoms and indices of biological aging (operationalized as shorter telomere length and greater mitochondrial DNA copy number [mtDNA-cn]). Specifically, we (Humphreys et al., 2020) showed that depressive symptoms at our baseline assessment when participants were, on average, 11.38 years of age, predicted higher rates of telomere erosion and greater increases in mtDNA-cn over a two-year period, over and above the effects of ELS; in contrast, markers of cellular aging at baseline did not predict subsequent changes in depressive symptoms. Thus, early depressive symptoms might affect the rate of cellular aging in children and adolescents. It will be important in future work to examine factors, such as social support, parenting behaviors, and exercise, that may buffer individuals from experiencing accelerated cellular aging, and to test the formulation that preventing depression will have long-term health benefits through reducing rates of telomere erosion and increases in mtDNA-cn. And as we describe below, we extended our study of biological aging to include the participants’ physical environments.

## An emerging focus on the physical environment

### Contaminants

Just over a decade ago when we were designing our study of the effects of ELS on trajectories of brain development, endocrinology, and clinical symptomatology over puberty, little attention had been paid to the effects of environmental pollutants and contaminants on symptoms of psychopathology, particularly in youth. Most studies of the effects of air and water pollution had focused on the physical health of adults, including cardiovascular disease (Brook et al., 2010; Navas-Aden et al., 2007), asthma (Clougherty et al., 2007), and Alzheimer’s Disease (Moulton & Yang, 2012). More recently, investigators have extended this focus to examine the effects of environmental toxins on mental health; in fact, the Environmental Protection Agency (EPA, 2023) and American Psychological Association (Clayton et al., 2023) have now published reports on the specific threat of climate change-related issues (e.g., extreme temperatures, natural disasters, etc.) to the physical and psychological health of developing children. Further, the WHO (2016) concluded that air pollution in particular is a major environmental threat to public health. Many of the health risks of air pollution have been attributed to fine particulate matter (PM2.5; air particles smaller than 2.5 *μ*m in diameter), which may be especially dangerous given its absorption into the bloodstream to reach the brain, and to ozone, a product of the interaction of pollutants with sunlight. In fact, epidemiological studies of adults are now linking exposure to ozone with depression-relevant phenomena, such as criminality (Lu et al., 2018) and antidepressant use (Zhao et al., 2018), and with suicides and emergency department visits for mental health (Biermann et al., 2009; Szyszkowicz et al., 2016).

Children and adolescents may be particularly vulnerable to the effects of ambient PM2.5 and ozone on brain development given the time they spend being physically active outdoors and, therefore, breathing in more air in proportion to their body weight than do adults. Indeed, researchers have begun to document negative effects of PM2.5 exposure on cognitive and affective functioning in youth (Jorcano et al., 2019; Peterson et al., 2015; Sunyer et al., 2015), although it is still unclear how air pollutants affect the brain. In this context, however, there is some evidence of pollution-related alterations in white matter in children (Burnor et al., 2021; Calderón-Garcidueñas et al., 2012; Peterson et al., 2015), as well as cortical thinning and reduced gray matter volume (Beckwith et al., 2020; Guxens et al., 2018).

When we designed our study, we had not considered examining how environmental toxins might interact with ELS to affect adolescent neurodevelopment. Recently however, given the increasing societal emphasis on climate change and its broad effects, combined with a growing body of research documenting the adverse effects of air and water pollution on psychobiological functioning, we began to explore the possibility of integrating measures of exposure to environmental toxins into our study. Doing so would allow us to examine the possible role of air and water contaminants in affecting relations between early adversity and trajectories of brain development and clinical symptoms in the children and adolescents in our project without adding to participant burden.

Jonas Miller, then a postdoctoral fellow in our lab, located an online resource, CalEnviroScreen, that was created by the Office of Environmental Health Hazard Assessment as a tool to map various environmental indicators for pollution and population characteristics across California neighborhoods at the census-tract level (Faust et al., 2017). CalEnviroScreen uses environmental, health, and socioeconomic information to produce scores for every census tract in the state. Most relevant to our project, CalEnviroScreen provides census-tract-level data for levels of PM2.5, ozone, and water quality, as well as neighborhood-level socioeconomic disadvantage, indexed by census-tract measures of poverty, educational attainment, unemployment, and housing burden. By mapping our participants’ addresses to CalEnviroScreen census-tract data, we are able to associate potential exposure to environmental toxins with sociodemographic variables and neurobiological functioning across adolescence.

In a series of studies using CalEnviroScreen data, we examined associations of PM2.5 with several of our constructs in our project. For example, Miller et al. (2019) found that adolescents residing in neighborhoods characterized by higher concentrations of PM2.5 had greater autonomic reactivity in response to social stress during a Trier Social Stress Test (TSST) and, further, that this association was moderated by symptoms of anxiety and depression, with youth who reported more severe anxiety and depression symptoms showing the strongest association between PM2.5 and autonomic reactivity. In a subsequent study using tensor-based morphometry, Miller et al. (2022) found that ELS moderated the effects of PM2.5 on changes in brain volume in distinct regions over a two-year period. More specifically, for adolescents who had experienced less severe ELS, PM2.5 was associated with volumetric changes across several gray and white matter regions; fewer effects of PM2.5 were observed for adolescents who had experienced more severe ELS. Thus, for several brain regions, exposure to ELS constrained the effects of PM2.5 on structural development.

In the context of biological aging that we discussed earlier, Miller et al. (2022) examined associations among pollution exposure, telomere length, and hippocampal volume. Miller et al. (2021) found that greater hippocampal volume exacerbated the association of neighborhood pollution burden with telomere length; further, in youth with larger hippocampal volume, pollution burden was indirectly associated with shorter telomere length approximately 2 years later through shorter telomere length at baseline, suggesting that living in high-pollution-burden neighborhoods predisposes children them to develop shorter telomeres later in adolescence.

Similarly, Uy et al. (under review) examined the association of pollution burden with brain functioning and found that community pollution burden was associated with greater activation and connectivity among brain regions within the default mode network during implicit emotion regulation of negatively-valenced stimuli, which in turn were associated with greater longitudinal increases in depressive symptoms in pollution-burdened adolescents. Finally, Uy et al. (in preparation) found that greater exposure to diesel particulate matter was associated with lower/blunted cortisol reactivity during the TSST, which was associated with more severe depressive symptoms in adolescents.

### Neighborhood disadvantage

There is also a growing focus in this field on the influence of relative socioeconomic (SES) advantage of an individual’s community on child development. As Hyde et al. (2020) highlight, the more immediate home environment (e.g., parenting behaviors, family discord) interacts with more distal neighborhood environment, and because children spend more time in the neighborhood as they mature, the neighborhood context becomes a more salient factor in shaping adolescent brain development. A growing literature has found neighborhood disadvantage is associated with mental health (Rakesh et al., 2021, 2023), cognition (Hackman et al., 2021), and brain structure (Miller et al., 2022; Rakesh et al., 2022), and function (Gard et al., 2021; Rakesh et al., 2021). In our lab, Dr Miller has integrated the data we collected from our participants with external databases to identify nuanced relations between the neighborhood SES disadvantage, cortical thickness, and depressive symptoms in adolescents (Miller et al., 2022). Specifically, we found that an overall thinner cortex mediated the association of greater neighborhood SES disadvantage with more severe depressive symptoms in adolescents, and that greater neighborhood SES disadvantage, over and above family SES, was uniquely associated with cortical thinning in regions implicated in emotion processing (e.g., superior frontal gyrus, insula, fusiform).

Recognizing that there are likely to be significant associations among neighborhood disadvantage, family SES, environmental contaminants, parenting behaviors, and overall health, we’ve begun to explore the associations among these variables and anxiety symptoms and white matter development in our sample of adolescents. For example, we (Buthmann et al., 2023) found that living in a community with greater SES disadvantage was associated with lower uncinate fasciculus structural integrity; however, if adolescents also reported higher parental warmth, anxiety symptoms were low. Moreover, when probing the aspects of the community that were associated with risk for poor structural integrity and anxiety symptoms, we identified poverty, contaminated drinking water, poorer health, and traffic density as important predictors. These findings underscore the importance of integrating environmental and neighborhood variables into the study of the neurobiology of adolescence in order to generate more comprehensive theories of the neural and behavioral development of youth.

## Inflammation

It is well documented that exposure to ELS is one of the strongest predictors of developing depression in adolescence (Heim & Binder, 2012; LeMoult et al., 2020). Indeed, we designed our project to increase our understanding of how adverse events “get under the skin” to increase risk for depression and other forms of emotional disturbance. Since we developed and began to conduct our project, research began to emerge implicating inflammation as a key causal pathway linking ELS and depression (Danese & J Lewis, 2017; Slavich & Irwin, 2014). For example, compared to non-maltreated children, maltreated children have been found to have higher peripheral levels of inflammatory biomarkers, such as C-reactive protein (CRP), interleukin-6 (IL-6), and tumor necrosis factor-α (TNF-α) (Copeland et al., 2014; Danese et al., 2011; Slopen et al., 2013). Importantly, adversity-related low-grade inflammation appears to persist across the lifespan; researchers have documented elevated concentrations of inflammatory markers in adults who, as children, were exposed to maltreatment and/or adversity (Danese et al., 2007, 2009; Matthews et al., 2014).

There is also now converging evidence that inflammation plays a causal role in the pathogenesis of depression. A recent meta-analysis of over 100 studies of cytokine levels in depression found that depressed patients had higher concentrations of inflammatory markers than did controls (Osimo et al., 2020). Other researchers have reported that concentrations of these inflammatory markers predict the subsequent development of depressive symptoms (Khandaker et al., 2014; Moriarity et al., 2019; Wium-Andersen et al., 2013). Thus, recent data suggest that inflammation is a psychobiological mechanism by which ELS can lead to depression; however, the neural mechanisms by which this process occurs are still unknown. We felt that it is important that we elucidate these mechanisms in light of the significant neurodevelopmental changes that characterize adolescent development. Given findings implicating inflammation in altered stress- and reward-related processing and depression (Pizzagalli et al., 2009), we were particularly well positioned to examine inflammation and brain function and structure in our study because of our RDoC focus on stress/affective and reward-related neural circuitry. By leveraging and integrating our existing data on ELS and our planned assessments and analyses of trajectories of neurodevelopment and depression, we were able to examine whether stress/affective and reward-related neurocircuitry is sensitive to the effects of inflammation and mediates the associations between ELS and depression and, further, to test whether the associations among inflammation, stress and reward circuitry, and depression depend on the types of ELS to which participants have been exposed.

At our third time point of data collection (2018–2021, 13–17 years of age), we began implementing a dried blood spot protocol to measure inflammation in our participants. This minimally invasive procedure yields a sample from which several cytokines and C-reactive protein (CRP) can be derived. From these data, Yuan et al. (2022) found that in the context of higher early life stress, higher CRP levels were associated with decreased ventro-lateral prefrontal cortex (vlPFC) activity and decreased amygdala-vlPFC functional connectivity during implicit regulation of negative emotional stimuli. Further, CRP was not associated with neural activation or connectivity in youth exposed to lower levels of early life stress, suggesting that ELS exposure disrupts neuro-immune signaling in emotion regulation circuitry. Recently, we also found that nucleus accumbens activity during reward processing was blunted in adolescents with higher levels of CRP and greater exposure to ELS (Yuan et al., 2023) and further, that a greater increase in CRP over two years was significantly associated with a smaller increase in amygdala volume, particularly in youth who experienced higher SES disadvantage (Yuan et al., under review).

## Sleep

An emerging literature has been documenting that sleep behaviors and quality undergo significant changes during the transition to adolescence, increasing the prevalence of sleep disturbances during adolescence (Crowley et al., 2018). These sleep disturbances have been linked to neural, endocrine, immune, and mental health outcomes during development, and are a prevalent feature of many physical and mental disorders. These sleep-related alterations in biology and in outcomes are similar to those that have been attributed to ELS; however, empirical investigations in these areas have been conducted in relative isolation (Fuligni et al., 2021). To increase our understanding of the role of sleep and its effects on the neural and biological mechanisms that link ELS to mental health outcomes during adolescence, we added several measures of sleep to our third assessment, including subjective reports of sleep time/quality, actigraphy recordings of sleep, and sleep diaries. In analyzing data obtained with these measures, we found that, consistent with formulations that pubertal processes underlie changes in sleep during adolescence, advanced pubertal stage, but not chronological age, was associated with poorer subjective sleep satisfaction, which in turn was associated with higher levels of negative affect (Kirshenbaum et al., 2023). Integrating these data with other measures of sleep that we had included earlier in the study, Uy and Gotlib (in press) also found that female adolescents who were exposed to more severe adverse events during childhood reported more sleep problems during early adolescence (9–13 years, our first timepoint) as well as greater longitudinal increases (across the first 3 time points) in sleep problems across adolescence, which in turn were each independently associated with longitudinal increases in depressive symptoms. Interestingly, for male adolescents, while more severe adverse events during childhood and sleep problems during adolescence were also related to increases in depressive symptoms, early adversity was not related to sleep problems, suggesting that there are sex differences in the mechanisms by which early adversity relates to depressive symptoms during adolescence. Uy et al. (2023) found, further, that more sleep problems were associated with lower uncinate fasciculus microstructural integrity in adolescents exposed to high levels of early adversity, and with higher cingulum cingulate integrity in adolescents exposed to low levels of early adversity, which in turn was associated with greater increases in depressive symptoms.

Finally, integrating measurements of environmental toxins, inflammation, and sleep, we found that greater pollution burden was associated with higher levels of CRP among adolescents who spent less time asleep (based on actigraphy). In contrast, pollution burden was not related to levels of CRP among adolescents who slept longer on average and also among those who engaged in greater catch-up sleep during the weekends, suggesting that extending sleep time (and/or catching up on lost sleep) may buffer the inflammatory effects of pollution (Uy et al., in preparation).

## COVID-19

We had designed our project initially to address the alarmingly high prevalence of ELS (Child Welfare Information Gateway, 2019; Kim & Drake, 2019) and its significant impact on mental health in youth. In analyzing the data we collected at the first two timepoints of our study, we were obtaining findings consistent with the formulation that ELS increases adolescents’ vulnerability or reactivity to the effects of stress, placing them at higher risk for developing symptoms of depression and other forms of emotional disturbance. Midway through the third timepoint assessment in March 2020, the global COVID-19 pandemic halted in-person research for what would prove to be almost a year. At the time, of course, we had no idea that the university would be closed for that long (and longer in other respects). We pivoted toward assessing in depth how the adolescents in our study were coping not with early life stress, but with a concurrent, multifaceted stressor. To adapt and ensure we could understand the toll of the global pandemic, we remotely administered several additional measures of loneliness, screen time usage, resilience, perceived stress, and other psychopathology symptoms (e.g., anxiety and obsessive-compulsive symptoms).

Amid a global backdrop of fear and uncertainty, the shelter-in-place orders in spring 2020 that led to school closures, academic disruptions, social restrictions, and reduced access to school-based mental health services (Golberstein et al., 2020) made the pandemic particularly difficult for children and adolescents (Jiao et al., 2020; Xie et al., 2020; Zhou et al., 2020). Indeed, a meta-analysis found that the prevalence of internalizing symptoms in children and adolescents doubled during the COVID-19 pandemic (Racine et al., 2021). We now know that, in addition to an enormous death toll, the pandemic led to increased personal and familial distress, economic hardship, and enforced social isolation (Galea et al., 2020; Holmes et al., 2020; Racine et al., 2021). We were well-situated to assess the extent of the psychological toll of the pandemic in our richly characterized sample.

Of course, we had not anticipated the pandemic when we designed our study, but we were nonetheless able to test the formulation that ELS would increase adolescents’ vulnerability to the effects of COVID-19-related stress, placing them at higher risk for developing symptoms of depression. As we expected, severity of ELS predicted levels of depressive symptoms during the pandemic, which continued to be higher in females than in males (Gotlib et al., 2021). Importantly, the association between ELS and depression was mediated by adolescents’ reported levels of perceived stress. In other studies, we used data we had obtained before the pandemic to predict our participants’ functioning during the lockdowns. For example, Chahal et al. (2021) found that early-maturing youth had greater increases in internalizing symptoms during the pandemic if the connectivity/coherence of their brains’ executive control network (ECN) at T1 was low; in contrast, relative pubertal stage was not associated with changes in internalizing symptoms in adolescents with higher ECN coherence, highlighting the role of neural functional architecture in protecting against risk factors that may exacerbate symptoms of internalizing psychopathology during periods of stress and uncertainty. Miller et al. (2021) reported similar predictive effects of pre-pandemic quality of parenting and connectivity between the amygdala and subgenual anterior cingulate cortex on subsequent depressive symptoms, and of pre-pandemic levels of family adversity and heart rate variability (Chahal et al., 2021).

When we resumed in-person research (and scanning), we also began to examine how adolescents who were assessed prior to the pandemic differed from adolescents who were assessed after the COVID-19 lockdowns ended. Prior to the pandemic, researchers found that exposure to ELS (i.e., violence, neglect, and family dysfunction) is associated with both poorer mental health and with maladaptive neurodevelopment indicative of accelerated brain maturation (Colich et al., 2020). Consistent with our hypotheses, we found that participants assessed after the pandemic began had higher levels of internalizing symptoms, reduced cortical thickness, larger limbic structures, and accelerated brain ages (Gotlib et al., 2023), underscoring the need for longitudinal studies that began before the pandemic to consider the effects of lockdowns on trajectories of neurodevelopment. Understanding factors that mitigate or exacerbate these impacts, as well as the continued developmental trajectories in a post-pandemic world, will continue to be an important part of our work.

## Implications of interdisciplinarity for training in developmental psychopathology

While it is easy to simply write here that interdisciplinarity is important (and, we think, necessary) for the advancement of developmental psychopathology and to encourage researchers to embrace this, we must recognize that a greater focus on interdisciplinarity has significant implications for training in this field. Fifty years ago, interdisciplinarity may have meant that investigators receive training that would allow them to integrate clinical psychology into the “academic” study of development and psychopathology. Today, the range and diversity of domains of research in developmental psychopathology seem limitless, arguably broader than a single investigator can master. As other investigators have described (e.g., Cicchetti & Toth, 2009; Susman et al., 2019), some of the challenges in conducting interdisciplinary research include (1) establishing communication and collaborations among researchers across disciplines, which may be especially challenging in institutions with traditional discipline-based academic departments; (2) bridging gaps in technical skills and knowledge between or among disciplines; (3) obtaining funding to conduct large-scale, interdisciplinary, longitudinal studies; and (4) finding and selecting suitable journals in which to publish interdisciplinary findings. Meeting these challenges will require us to ensure that the next generations of scientists have the opportunity to receive interdisciplinary training in order to conduct the most impactful research possible in developmental psychopathology.

In considering the changes that such training would entail, it is clear that there are not only advantages over current training models, but also drawbacks and obstacles to receiving training aimed at increasing the interdisciplinarity of trainees. For example, as trainees are taking courses with experts across disciplines to broaden their understanding of various aspects of developmental psychopathology, they may at the same time be sacrificing depth of their knowledge and skills. And with this training, graduate degrees and postdoctoral fellowships may require more mentors and additional time to complete, which can place a burden on and exacerbate disparities for those trainees who are experiencing economic hardship. Certainly, funding opportunities are available – for example, the NIH’s K-Award mechanism – but they remain extremely competitive. And compounding this situation, the more interdisciplinary a training proposal becomes, the more difficult it will be to find expert reviewers (also an issue, of course, with interdisciplinary scientific papers!).

We should note that there is already an increasing demand and expectation that trainees in developmental psychopathology develop skills in advanced methodologies and statistical techniques. The emergence both of sophisticated statistical approaches to data analysis, such as structural equation modeling, k-means clustering, and machine learning, and of larger longitudinal, multivariate, publicly available datasets can both help trainees to begin to engage in interdisciplinary research and hinder the depth of theoretical knowledge that they are able to develop. And as a related point, larger sample sizes and the emerging need for reproducibility of findings extract a considerable toll in time, funding, and resources.

At the institutional level, interdisciplinary collaborations will require financial support, space, supervision, and other resources that may be limited. Expectations of what constitutes a “focus” of a research program for tenure decisions will have to change, as will valuations of single- versus multiple-authored scientific papers. As a related point, current standards of “productivity” for investigators may have to be revisited in light of the time and effort it takes to develop and foster interdisciplinary collaborations, collect multi-domain data, and prepare specialized grant applications and manuscripts that bridge more than one discipline. By making explicit efforts to address these hurdles and difficulties, institutions and funding bodies can increase both the number of interdisciplinary trainees and investigators, and the quality of boundary-breaking research. As Susman et al. (2019) note, we can begin to address challenges in this area by encouraging communication, terminology, and research practices across disciplines, facilitating cross-discipline data sharing, and beginning interdisciplinary training at the undergraduate level.

## Concluding comments

As our understanding of the brain, the body, and the environment has advanced, so too has the field of developmental psychopathology. It has become increasingly apparent over the past few decades that the emergence of psychopathology, often in the face of adversity, cannot be studied in isolation because it does not develop independent of many other personal and environmental factors. To gain expertise in developmental psychopathology now requires researchers to be open and flexible, and to be knowledgeable about a range of domains, from dynamic and interacting biological systems to equally dynamic and interacting environments in which individuals live. Research that focuses on developmental psychopathology without taking the perspective of a different (even if related) discipline is not likely to move the field forward in a significant way. Indeed, working towards the goals that Masten (2006) formulated – to prevent and alleviate the suffering of psychopathology – we have made great strides in identifying mechanisms through which a range of adverse experiences can affect children’s and adolescents’ psychobiological and emotional development. These discoveries, and those still to come as our field continues to evolve, are contributing to the generation of prevention and intervention strategies that can reduce the burden of psychopathology on individuals, families, and society. Supporting and promoting strong interdisciplinary research is paramount to seeing these goals to fruition.
